# Complete response of renal cell carcinoma vena cava tumor thrombus to neoadjuvant immunotherapy

**DOI:** 10.1186/s40425-019-0546-8

**Published:** 2019-03-11

**Authors:** Craig Labbate, Ken Hatogai, Ryan Werntz, Walter M. Stadler, Gary D. Steinberg, Scott Eggener, Randy F. Sweis

**Affiliations:** 1 Section of Urology, Department of Surgery, University of Chicago, Chicago, IL USA; 2 Section of Hematology/Oncology, Department of Medicine, University of Chicago, Chicago, IL USA

**Keywords:** Neoadjuvant immunotherapy, Ipilimumab, Nivolumab, Tumor thrombus, Nephrectomy, Thrombectomy, Renal cell carcinoma

## Abstract

**Background:**

Clinically localized renal cell carcinoma is treated primarily with surgery followed by observation or adjuvant sunitinib in selected high-risk patients. The checkpoint inhibitor immunotherapeutic agents nivolumab and ipilimumab have recently shown a survival benefit in the first-line metastatic setting. To date, there have been no reports on the response of localized renal cancer to modern immunotherapy. We report a remarkable response of an advanced tumor thrombus to combined immunotherapy which facilitated curative-intent resection of the non-responding primary renal tumor. We characterized the tumor microenvironment within the responding and non-responding tumors.

**Case presentation:**

A 54-year-old female was diagnosed with a locally advanced clear cell renal cell carcinoma with a level IV tumor thrombus of the vena cava. She was initially deemed unfit for surgical resection due to poor performance status. She underwent neoadjuvant immunotherapy with nivolumab and ipilimumab with a complete response of the vena cava and renal vein tumor thrombus, but had stable disease within her renal mass. She underwent complete surgical resection with negative margins and remains disease-free longer than 1 year after her diagnosis with no further systemic therapy. Notably, pathologic analysis showed a complete response within the vena cava and renal vein, but substantial viable cancer remained in the kidney. Multichannel immunofluorescence was performed and showed marked infiltration of immune cells including CD8^+^ T cells and Batf3^+^ dendritic cells in the thrombus, while the residual renal tumor showed a non-T cell-inflamed phenotype.

**Conclusions:**

Preoperative immunotherapy with nivolumab and ipilimumab for locally advanced clear cell renal cancer resulted in a complete response of an extensive vena cava tumor thrombus, which enabled curative-intent resection of a non-responding primary tumor. If validated in larger cohorts, preoperative immunotherapy for locally advanced renal cell carcinoma may ultimately impact surgical planning and long-term prognosis.

## Background

Vascular invasion with tumor thrombus may occur in advanced renal cell carcinoma (RCC) and other tumors, such as hepatocellular and adrenocortical carcinoma. The natural history of untreated patients with RCC tumor thrombus is poor if aggressive surgical resection is not feasible [1]. Aggressive surgical resection of an advanced tumor thrombus (level III/IV) results in about a 50% five-year cancer specific survival in the absence of metastatic disease [2–4]. While there is a potential survival advantage with aggressive surgical resection with thrombectomy, it carries significant potential morbidity and mortality.

Immunotherapy with checkpoint inhibitors is now a standard treatment for metastatic RCC. Nivolumab, a programmed cell death 1 (PD-1)-inhibitor, was initially approved for second-line treatment after antiangiogenic therapy. The CheckMate 025 study in this setting demonstrated improved overall response rate (ORR) and overall survival (OS) compared to everolimus [5]. Combination therapy with anti-PD-1 and anti-CTLA-4 immune checkpoint inhibitors had been successful in metastatic melanoma and was subsequently explored in metastatic RCC. The CheckMate 214 trial compared the combination of nivolumab and ipilimumab against sunitinib in treatment-naïve patients with metastatic RCC. The combination therapy demonstrated improved OS when compared to sunitinib (HR = 0.63) in patients with intermediate- and poor-risk disease by International Metastatic RCC Database Consortium (IMDC) classification. Additionally, a significant improvement in the ORR (42% vs. 27%) was observed, including 9% of patients achieving a complete response in the combination immunotherapy arm versus 1% with sunitinib. This trial led to the Food and Drug Administration (FDA) approval of nivolumab in combination with ipilimumab for first-line IMDC intermediate or poor-risk metastatic RCC.

There are currently no FDA-approved neoadjuvant systemic treatments for patients with localized renal cancer. Multiple tyrosine kinase inhibitors (TKIs) have been evaluated in patients with locally advanced disease with the objective of downstaging to allow surgical resection. Case series and phase II trial data have shown low rates of response [6]. In patients with a tumor thrombus, the data is sparse. In one study of 25 patients with tumor thrombus from RCC, neoadjuvant TKI therapy reduced the thrombus level in only 12% of patients and altered the surgical approach in only 1 patient [7]. Another smaller study showed similarly low efficacy of TKI treatment, with reduction of the thrombus level in only 1 of 14 patients [8]. We report a case of a profound response of an RCC tumor thrombus to combined immunotherapy with ipilimumab and nivolumab, with radiographic and immunopathologic signs of tumor resistance in the primary kidney tumor.

## Case report

**Fig. 1:**
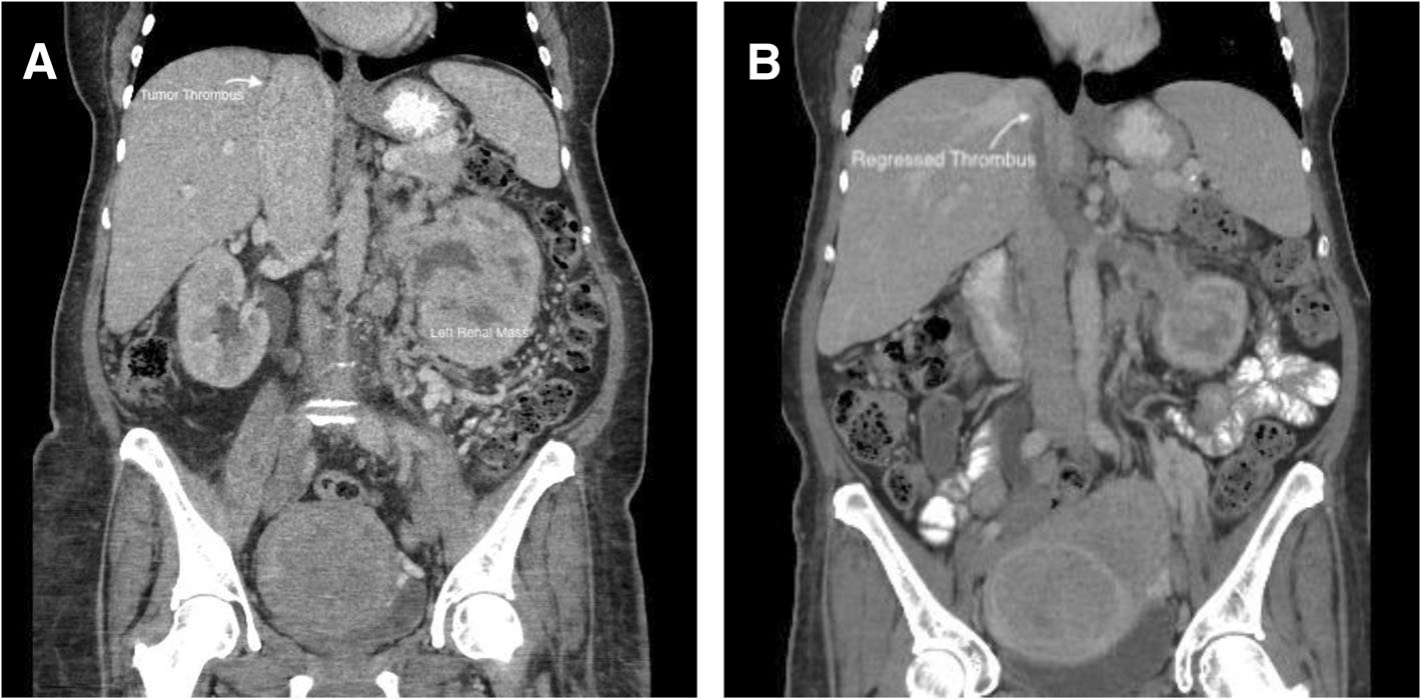
Coronal images of tumor thrombus pre- (**a**) and post- (**b**) immunotherapy

A 58-year-old female presented with 40-pound weight loss over several months with new lower extremity edema, dyspnea on exertion, and flank pain. A CT scan revealed a 12.2 cm × 8.1 cm left renal mass with regional adenopathy and a bulky thrombus extending cephalad within the inferior vena cava (IVC) to the junction of the IVC and right atrium with occlusion and distension of the IVC with maximum thrombus diameter of 49 mm (Fig. 1a). Upon contrast administration, the thrombus showed strong uniform enhancement, confirming suspicion of tumor thrombus. No bland thrombus was identified, so anticoagulation was not initiated. A transthoracic echocardiogram revealed no tumor within the right atrium. Serum hemoglobin was 7.8 g/dL, corrected calcium level was 9.8 mg/dL, absolute neutrophil count was 9.88 × 10^9^/L and platelet count was 474,000/uL. She underwent a CT of the chest and MRI of the brain without evidence of metastasis. A core needle biopsy of the renal mass showed clear cell renal cell carcinoma, WHO/ISUP grade 3 with focal grade 4 and with no identified sarcomatoid elements.

Her Eastern Cooperative Oncology Group (ECOG) performance status was 3, so she was deemed not a surgical candidate. Given her IMDC poor-risk classification, she was offered systemic immunotherapy with the possibility of consolidative surgery if she had a radiographic response. She received 4 cycles of nivolumab and ipilimumab and her re-staging CT revealed stable locoregional adenopathy; the renal mass slightly decreased to 9.1 cm in diameter. The thrombus, however, had regressed from the cavo-atrial junction to the suprahepatic IVC with a marked decrease in diameter so that it no longer obliterated the IVC (Fig. 1b). She then received 4 cycles of nivolumab monotherapy dosed 480 mg every 4 weeks. She had marked improvement in her ECOG performance status to 1 and resolution of lower extremity edema and dyspnea. A second follow up CT re-demonstrated the regressed and non-enhancing tumor thrombus, with persistence of the primary renal mass, which measured 10.4 cm.

She subsequently underwent a left radical nephrectomy and IVC thrombectomy through a chevron incision. There were dense adhesions near the renal hilum and bulky lymphadenopathy which required *en-bloc* ligation of the hilum. Hilar and para-aortic lymph node sampling was performed. The tumor thrombus remnant was estimated to be 5 mm in diameter. After obtaining proximal and distal vascular control, the vena cava was entered at the renal vein ostium. A long, thin, firm, intravascular thrombus was encountered, which was densely adherent to the endothelium without a discernable surgical plane It was deemed unable to be extracted without resection of a substantial portion of the sub-diaphragmatic vena cava. Samples were sent to pathology. The renal vein and vena cava cuff were resected and reconstructed with running non-absorbable suture. Her post-operative course was uneventful. All systemic therapy was discontinued after surgery and she remains without evidence of disease longer than 1 year after her original diagnosis.

**Fig. 2:**
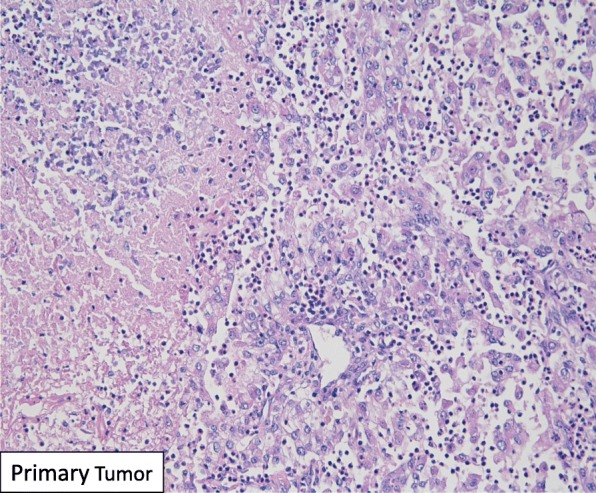
H&E staining of remaining viable renal tumor with a dense neutrophilic infiltrate after immunotherapy

Final pathologic analysis revealed a 6.3 cm ISUP Grade III clear cell renal cell carcinoma with focal rhabdoid features (5%) and sinus fat invasion of the left kidney. The primary tumor demonstrated areas of necrosis as well as a dense neutrophilic infiltration alongside viable tumor without evidence of treatment response (Fig. 2). The resected residual renal vein thrombus was characterized by hemosiderin-laden macrophages and other signs of treatment effect, but no viable tumor was present within the IVC cuff or main renal vein. There was viable tumor thrombus present within segmental renal veins of the renal sinus. The 13 sampled regional lymph nodes had no evidence of carcinoma or treatment effect to suggest any previous malignant infiltration.

**Fig. 3:**
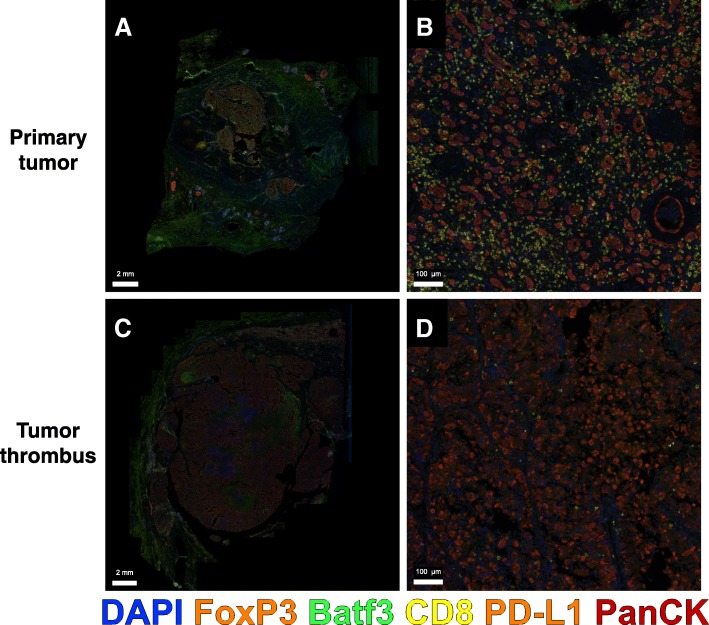
Multichannel immunofluorescence of renal mass and tumor thrombus. Representative images of residual tumor in the segmental renal vein that responded to therapy at low power (**a**) and high power (**b**) with clusters of co-localized CD8^+^ T cells and Batf3^+^ dendritic cells. The primary renal tumor staining pattern is shown at low power (**c**) and high power (**d**) featuring far fewer Batf3^+^ cells and CD8^+^ T cells

PD-L1 immunohistochemistry on the renal tumor showed absence of staining in most of the tumor. Subsequently, multichannel immunofluorescence for Pan CK, CD8, PD-L1, FoxP3, Batf3, and DAPI was performed on the residual renal tumor and remaining segmental renal vein tumor using the PerkinElmer Vectra Polaris system (Fig. 3). The primary renal tumor appeared to be immune-excluded and lacked infiltration of CD8^+^ T cells or Batf3^+^ dendritic cells. In contrast, within the residual segmental renal vein tumor thrombus, we observed a marked infiltration of CD8^+^ T cells, FoxP3^+^ regulatory T cells, and Batf3^+^dendritic cells. The non-inflamed renal tumor lacked PD-L1 expression whereas the tumor thrombus remnant showed interspersed strongly positive PD-L1 expressing cells in stromal areas (Fig. 3b).

## Discussion

We describe a patient who initially presented with locally advanced RCC and level IV vena cava thrombus with regional lymphadenopathy, poor performance status, and severe lower extremity edema. She was started on nivolumab and ipilimumab combination therapy and had a complete pathologic response within the tumor thrombus of the IVC and renal vein with radiographically stable disease within the kidney. Immunotherapy was well-tolerated and resulted in vast improvement in performance status which permitted consolidative surgical therapy with curative intent. The patient remains disease-free while off all systemic therapy Clinical trials have generally excluded patients with poor performance status, thus evidence to support immunotherapy in a patient such as this one has been lacking. To our knowledge, this case is the first reported complete IVC tumor thrombus response to preoperative immunotherapy in renal cell carcinoma and highlights variable immunological responses in the primary tumor and tumor thrombus.

Case reports with complete responses of T3b renal cell carcinoma to neoadjuvant therapy have been reported in the TKI era [9]. Larger case series, unfortunately, have shown that meaningful tumor thrombus regression defined by either downstaging or favorably changing the surgical approach is uncommon [8]. This case suggests outcomes may be improved in this setting in the era of immunotherapy. Treatment with ipilimumab and nivolumab shrank the tumor thrombus in both extension and diameter, downstaging clinically from Mayo level IV to level III. More importantly, the thrombus decreased in diameter from 49 mm to 5 mm thus returning normal IVC physiology and improving the patient’s functional status. This allowed the patient, who was previously not a surgical candidate, to receive curative surgical therapy. While tumor did remain within the segmental veins of the renal sinus, the complete tumor response in the sampled IVC and renal vein downstaged the patient to a pathologic Mayo thrombus level 0, and allowed resection with negative margins potentially with radical nephrectomy alone. Combination immunotherapy with PD-L1 and CTLA-4 inhibitors has shown much higher complete and overall response rates than TKIs in the metastatic setting [10]. As a result, multiple phase II/III trials are now evaluating the survival benefit of perioperative immunotherapy [11].

Previous VEGF-targeted therapy with TKIs has been associated with increased local wound complications [12]. In this case, we report a difficult intraoperative dissection, potentially due to post-immunotherapy inflammation. Nonetheless, the operation was completed safely with minor adjustments and without postoperative complication. These findings are consistent with prior literature involving the safety of laparotomy after ipilimumab [13]. Notably the post-immunotherapy thrombus was densely adherent to the vessel wall and required a change in intraoperative decision making. These dense adhesions may be associated with tumor response and should be evaluated in future studies with neoadjuvant immunotherapy. Another unique finding in this case was the discrepancy between final radiographic diameter (10.4 cm) and pathologic tumor diameter (6.4 cm). In general, CT maximum diameter correlates with maximal pathologic diameter, though overestimates up to 5 cm have been reported [14]. Immunotherapy can occasionally cause radiographic overestimation of tumor size, or pseudoprogression, due to edema and tumor-infiltrating immune cells [15].

A likely reason for the dramatic response in the IVC thrombus and not the primary kidney mass is heterogeneity in the tumor immune microenvironments. The tumor microenvironment is a dynamic interaction between tumor cells, immune cells, extracellular matrix, and various stromal cells which can facilitate or inhibit tumorigenesis and immune evasion [16]. Programmed death ligand 1 (PD-L1), a T cell inhibitory molecule, is upregulated in response to interferon gamma and associated with a T cell-inflamed tumor microenvironment. In RCC, the role of PD-1/PD-L1 staining to predict tumor response to immunotherapy is still an evolving field. Elevated PD-L1 expression on renal biopsy is associated with worse overall survival, but also a higher rate of response to immunotherapy in metastatic disease [5, 14]. PD-L1 has recently been shown to be expressed differently by location in RCC. In 39 treatment-naïve patients with primary renal tumors and tumor thrombi, PD-L1 expression was rarely uniform and was much more common in the primary tumor (56%) than in the tumor thrombus (10%) [17]. Additionally, the presence of dendritic cells in RCC tumors has been associated with better prognosis [18]. In our patient, the tumor thrombus remnant in the segmental vein showed a higher expression of PD-L1 in association with other immune cells including CD8^+^ T cells and Batf3^+^ dendritic cells. On the contrary, the remaining viable tumor within the kidney, lacked PD-L1 expression or the presence of tumor-infiltrating immune cells. Combining these data, the tumor thrombus appeared to have a T cell-inflamed tumor microenvironment compared with the primary tumor, which was non-T cell inflamed. This heterogeneity likely explains the differential responses to immunotherapy in this case.

There are some limitations to this analysis. Primarily, the segments of tumor thrombus that had completely responded to therapy could not be evaluated, since no residual tumor was present at the time of surgery. Instead, our analyses on the segmental vein thrombus are assumed to represent the regressing vena cava tumor thrombus. Additionally, there may be different additional unmeasured secondary escape pathways after anti-PD-1 therapy, such as adenosineA2A overexpression or TGFβ production, independent of PD-L1, which may account for differential responses [19].

A second potential biomarker of differing immune responses in this case was neutrophilic infiltration. There were neutrophilic infiltrates within the T cell-excluded renal primary tumor, but these were not noted in the tumor thrombus. Tumor-infiltrating neutrophils have been associated with poor prognosis and upregulation of the VEGF pathway in RCC [20, 21]. VEGF activation has in turn been associated with impaired T cell trafficking to the tumor and impairment of the anti-tumor immune response [22, 23]. In addition, systemic measurement of a peripheral neutrophil-lymphocyte ratio indicates that high values after immunotherapy have correlated with poor prognosis [24].

In addition to the different T cell and neutrophil localizations suggestive of different tumor microenvironments, there are other possible mechanical explanations for the renal thrombus’s different response. Features within the primary tumor, such as intratumoral acidosis, hypoxia and increased interstitial pressure caused by aberrant angiogenesis are known to inhibit the immune response [16]. In this case, the tumor thrombus may be different from the primary tumor in regard to these mechanical and physiologic features, leading to a stronger immune response.

## Conclusions

We present a case of locally advanced RCC with a complete response within the tumor thrombus of the IVC and renal vein to preoperative nivolumab plus ipilimumab, which facilitated curative-intent resection of the non-responding primary renal tumor. In this case, the tumor thrombus showed evidence of a T cell-inflamed tumor microenvironment with co-localization of Batf3^+^ dendritic cells and CD8^+^ T cells and patchy expression of PD-L1, whereas the immunotherapy-resistant primary renal tumor showed T cell exclusion without PD-L1 expression. Surgical resection of advanced tumor thrombus in renal cell carcinoma is technically challenging and associated with added surgical morbidity. Thus, this case supports further investigation into preoperative combined immunotherapy, with the intent to facilitate curative surgical resection in patients with locally advanced RCC with tumor thrombus.

## Abbreviations

CTLA4: cytotoxic T-lymphocyte-associated protein 4
IMDC: International Metastatic RCC Database Consortium
ORR: overall response rate
OS: overall survival
PD1: programmed cell death protein 1
PD-L1: programmed cell death ligand 1
RCC: renal cell carcinoma
TKI: tyrosine kinase inhibitor
